# The Comparison of Lower Extremity Length and Angle between Computed Radiography-Based Teleoroentgenogram and EOS^®^ Imaging System

**DOI:** 10.3390/diagnostics12051052

**Published:** 2022-04-22

**Authors:** Kwang-Rak Park, Jae-Ho Lee, Dae-Soo Kim, Ho Ryu, Jaeho Kim, Chang-Jin Yon, Si-Wook Lee

**Affiliations:** 1 Department of Anatomy, Keimyung University College of Medicine, Daegu 42601, Korea; airboba@naver.com (K.-R.P.); anato82@dsmc.or.kr (J.-H.L.); 2 CTO-MAiT Co., Ltd., Daegu 42061, Korea; dskim@wkit.co.kr; 3 Industry-Academic Cooperation Foundation, Keimyung University, Daegu 42601, Korea; r7826974@gmail.com; 4 Dongsan Medical Center, Department of Orthopedic Surgery, School of Medicine, Keimyung University, Daegu 42601, Korea; zclass94@naver.com (J.K.); poweryon@nate.com (C.-J.Y.)

**Keywords:** computed radiography-based teleoroentgenogram, EOS^®^ imaging system, limb length, angular deformity

## Abstract

Background and objectives: The differences between computed radiography-based teleoroentgenograms (CR-based teleoroentgenograms) and an EOS^®^ imaging system were evaluated by measuring lower extremity lengths and alignments. Materials and methods: The leg length [L], femur length [F], tibia length [T], and hip–knee–ankle (HKA) angle were measured in 101 patients with lower extremity disease by a CR-based teleoroentgenogram with computed radiography and an EOS^®^. The additive length of the femoral and tibial segments (F + T) was determined by adding the two length values. Then, the differences among all five parameters between the two techniques were analyzed. The magnification (mm) was calculated by subtracting the length measurements on the EOS^®^ from those in the scanogram. Furthermore, the magnification percentage (%) was calculated by dividing the magnification with the measurements on the EOS^®^. Results: The magnification errors (mean ± standard deviation), when comparing both right and left sides, were 7.80 ± 1.41%, 7.3 ± 6.01%, 5.16 ± 1.25%, and 6.45 ± 0.94% for L, F, T, and F + T, respectively. For limb length, the CR-based teleoroentgenogram had an average magnification of 6.8% (range, 5.2 to 7.8%) compared to the EOS^®^ imaging. The two groups displayed a statistical difference (*p* < 0.01), except for the HKA angle. Conclusions: The CR-based teleoroentgenogram had a magnification of about 6.8% compared to the EOS^®^ imaging system in evaluating lower extremity length. Therefore, more attention must be given to CR-based teleoroentgenograms to correct angular deformities.

## 1. Introduction

Limb-length discrepancy (LLD) and angular deformity of lower extremities are relatively common in the general population [1]. These are especially prevalent in patients with spine and lower extremity disorders [2]. Although the impact of limb-length discrepancy and angular deformity on long-term functional outcome and health-related quality of life is uncertain, they are still worth noting. These can cause gait deviations and affect the function and longevity of joints in the lower extremity and lumbar spine [3,4,5,6]. The diagnosis of limb-length discrepancy and angular deformities usually begins with history-taking and physically examining the patient. However, confirming and accurately quantifying these disorders requires reliable imaging studies [4]. Patients with or at risk for clinically noticeable LLD or angular deformities often require multiple radiologic studies. Utilizing the appropriate clinical information and imaging studies is essential before making a treatment strategy.

Although various techniques, including ultrasound and computed tomographic scanning, are currently being utilized to evaluate LLD, plain radiography is the most reasonable method [7,8,9]. The three commonly used plain radiographic techniques are a variation of a teleoroentgenogram, an orthoroentgenogram, and a scanogram [7]. A teleoroentgenogram procedure uses a single radiographic exposure to both lower limbs, with the X-ray beam fixed at the center of the knee. On the other hand, three distinct exposures centered over the hip, knee, and ankle are used in an orthoroentgenogram procedure to minimize measurement errors secondary to magnification. In addition, the scanogram is a modification of the orthoroentgenogram, taken with three separate exposures centered at the hip, knee, and ankle using a standard-sized cassette. However, these general radiography techniques suffer from errors due to magnification and image stitching and have the disadvantage of requiring longer cassettes [7,10,11]. Currently, the most widely used modified teleoroentgenogram uses three radiographic exposures with digitally stitched-in computed radiography, called “computed radiography-based teleoroentgenogram (CR-based teleoroentgenogram).” 

The more recently developed EOS^®^ 3D biplanar radiograph (3D BR) imaging system is a novel low-dose biplanar digital radiographic imaging system that uses an ultrasensitive multiwire proportional chamber detector to detect X-rays. It simultaneously takes anteroposterior (AP) and lateral 2D images of the whole body in a calibrated environment, permitting 3D reconstruction. The advantage of EOS^®^ imaging includes true-to-size images of appropriate quality, delivered by low radiation doses based on other studies [12]. Because the EOS^®^ machine scans the body with two 45 cm-wide X-ray beams and the patient-to-beam distance is much closer, true-to-size images can be taken. Compared to a single-source divergent X-ray beam in conventional radiology, the patient-to-beam distance is relatively long, causing magnification of the images. Several studies reported on a 5–8% magnification between conventional radiographs. Thus, EOS^®^ imaging is most useful for minimizing limb-length discrepancy, sagittal imbalance, and angular deformity [7,11,13]. 

Current scientific literature has yet to report the magnification difference between CR-based teleoroentgenogram and EOS^®^ imaging. The purpose of this study is to evaluate the differences in lower limb length and the hip–knee–ankle (HKA) angle between the standing CR-based teleoroentgenogram and EOS^®^ imaging.

## 2. Materials and Methods

This study was conducted between April 2019 and October 2020 with 101 patients (56 males, 45 females) with diseases requiring lower limb alignment evaluation (Table 1). First, the patients who underwent the CR-based teleoroentgenogram test visited the hospital for follow-up treatment, and the EOS^®^ imaging system test was performed to confirm the improvement of the disease. After explaining the use of CR-based teleoroentgenogram images and the EOS^®^ imaging system test to the patients, this study was conducted with the patients who submitted informed consent. This study was conducted as a prospective and retrospective cohort study and was approved by the Institutional Review Board (IRB No. 201910003).

### 2.1. CR-Based Teleoroentgenogram 

**Figure 1:**
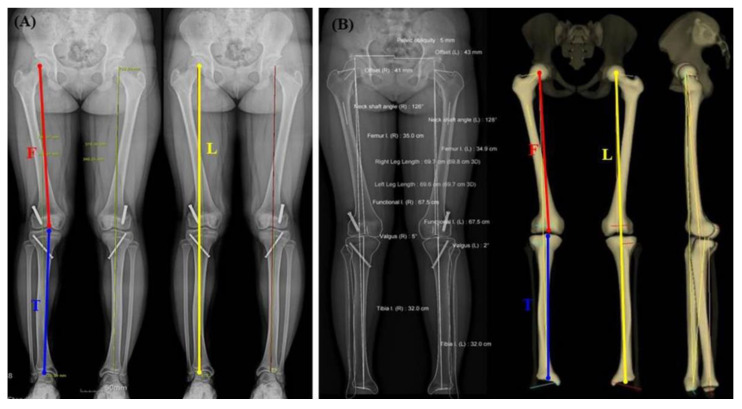
(**A**) Standing anteroposterior radiograph of the lower extremities on the CR-based teleoroentgenogram, demonstrating measurements of the leg (L), femur (F) and tibia (T) lengths, and hip–knee–ankle angle (HKA). (**B**) Standing anteroposterior and lateral radiograph of the lower extremities on EOS^®^, demonstrating measurements of the leg (L), femur (F) and tibia (T) lengths, and hip–knee–ankle angle (HKA).

The anteroposterior standing radiographs of the lower extremities (modified teleoroentgenogram) were taken using an X-ray tube assembly (Shimadzu Corporation, Kyoto, Japan) at a source-to-image distance of 200 cm at 70–150 kVp and 30–160 mAs with the patient facing the radiographic tube and both patellae facing forward. The X-ray tube location was fixed with a movement angle of 5–10°. It used three radiographic exposures centered at the hip, knee, and ankle using a digital moving cassette (46 cm × 46 cm) (Medlink Imaging, Pine Brook, NJ, USA). The radiographic images were retrieved using the picture archiving and communication system (PACS) software (M6, INFINITT Healthcare, Seoul, Korea), where radiographic measurements were performed. All measurements of the CR-based teleoroentgenogram were performed independently by three experienced orthopedic surgeons on a PASC workstation. The average values of the measured values were used for statistical analysis (Figure 1A).

### 2.2. EOS^®^

The patients were positioned identically to the modified teleoroentgenogram procedure. Two vertically moving X-ray beams scanned in a perpendicular plane. Scans were performed from the umbilical to the plantar level to show the entire lower limb in one take using 85 kV and 20 mA parameters. The acquired two-plane images were converted into a 3D model using the dedicated software, SterEOS^TM^ (EOS Imaging, Paris, France). All measurements on EOS^®^ images were independently performed by three experienced orthopedic surgeons on a SterEOS^®^ workstation. The average of each measurement value was used for statistical analysis (Figure 1B).

### 2.3. Radiographic Measurement

Functional leg, femur and tibia lengths, and also the hip–knee–ankle angle were measured using the CR-based teleoroentgenogram and EOS^®^ imaging. Leg length (L) dimensions were taken from the center of the femoral head to the top of the talus. For the femur length (F), measurements were obtained from the top of the intercondylar notch to the center of the femoral head. Lastly, the tibia length (T) was determined from the top of the talus to the center of the intercondylar eminence. The femoral and tibial segment (F + T)’s additive length was determined by adding the femoral and tibial lengths. The hip–knee–ankle (HKA) angle was defined as the angle between the mechanical axis of the femur and the tibia. 

Magnification (mm) was calculated by subtracting the length measurements obtained in the EOS^®^ from those in the CR-based teleoroentgenogram. Correspondingly, the magnification percentage was calculated as shown in the formula below:

$$\mathrm{Magnification}\;\mathrm{percentage}\;(\%)=\left[\frac{\mathrm{Measurements}\;\mathrm{in}\;\mathrm{CR}-\mathrm{based}\;\mathrm{teleroentgenogram}}{\mathrm{Measurements}\;\mathrm{in}\;{\mathrm{EOS}}^{®}}\right]-1\times 100\%$$

### 2.4. Statistical Analysis

T&F program version 3.0 (YooJin BioSoft, Seoul, Korea) was used for all statistical analyses. Data were expressed as mean ± standard deviation for continuous variables according to the normality. For categorical variables, the sample number and percentage were computed. A paired T-test between the CR-based teleoroentgenogram and the EOS^®^ was performed to analyze all five categories: L, F, T, F + T, and HKA angle. The significance of the statistical test was defined at a *p*-value < 0.05.

## 3. Results

The investigated data of the Intra-observer Interclass Correlation Coefficients (ICCs) of the EOS^®^ were 0.99 for leg, femur, and tibia lengths, respectively. Furthermore, the inter-observer ICCs of Stitch CR were 0.95, 0.96, and 0.95, respectively.

### 3.1. Right-Side Comparison

The results of the right-side limb-length and angle measurements, including the magnification and magnification percentages, are presented in Table 2. Compared to the CR-based teleoroentgenogram, the magnification values with the application of the EOS^®^ were 8.01% (53.7 mm) for L, 8.44% (30.45 mm) for F, 5.12% (15.75 mm) for T, and 6.85% (46.2 mm) for F + T. In all four categories, the magnification obtained from the CR-based teleoroentgenogram was significant (*p* < 0.01). On the other hand, the HKA angle did not show a statistical difference between the CR-based teleoroentgenogram and EOS^®^ imaging (*p* = 0.181).

### 3.2. Left-Side Comparison

The data for the left-side limb-length and angle measurements, including the magnification and magnification percentages, are displayed in Table 3. The magnification values with the application of the EOS^®^, in comparison to the CR-based teleoroentgenogram, were 7.61% (51.13 mm) for L, 6.81% (23.99 mm) for F, 5.20% (15.99 mm) for T, and 6.05% (44.34 mm) for F + T. In these four categories, the magnification from the CR-based teleoroentgenogram was significant (*p* < 0.01). Furthermore, the HKA angle did not show a statistical difference between the CR-based teleoroentgenogram and EOS^®^ imaging (*p* = 0.66).

### 3.3. Comparison for Both Sides 

The findings for both the right and left limb-length and angle measurements, including the magnification and magnification percentages, are shown in Table 4. Compared to the CR-based teleoroentgenogram, the magnification data with the application of the EOS^®^ were 7.80% (52.42 mm) for L, 7.63% (27.21 mm) for F, 5.16% (15.87 mm) for T, and 6.45% (3.09 mm) for F + T. The overall magnification in these four categories for the CR-based teleoroentgenogram was significant (*p* < 0.01). On the other hand, the HKA angle did not show a statistical difference between the CR-based teleoroentgenogram and EOS^®^ imaging (*p* = 0.177).

## 4. Discussion

A radiographic assessment of LLD must balance the need for the accurate assessment of limb length with the risks associated with repeated radiation exposure. Accuracy refers to the deviation between the measured value obtained with various techniques and the actual value of the bone length. In comparison, reliability is the deviation of values measured among different observers or multiple measurements by a single observer [14]. Accurate evaluation of limb-length discrepancy and HKA deformity plays an important role not only in surgical decision but also in maintaining appropriate alignment after surgery [15]. 

Previously, many authors reported the results of LLD measurements using plain X-ray, CT, MRI, and ultrasound equipment [8,9,16]. However, when selecting an inspection method, problems such as convenience, accessibility, reliability, accuracy, and radiation dose must be considered. Although computed tomography scanning is relatively accurate in measuring limb length, its daily use is limited due to low accessibility, inability to perform weight-bearing tests, and high radiation dose. 

Previous studies presented radiographic techniques for evaluating various types of LLD, such as orthoroentgenograms, teleoroentgenograms, slit-scanograms, and microdose digital radiography [3,5,17,18]. These techniques have the potential disadvantages of handling the longest films and cassettes and requiring specialist equipment, such as grids and filters. However, conventional teleoroentgenograms are widely available because they involve a single brief exposure and are less prone to motion artifacts. Among conventional radiographs, more recent advances in digital image acquisition and processing allow for the creation of similar full-length images from multiple smaller images that are digitally “stitched” together. One of these is the previously mentioned CR-based teleoroentgenogram, a method associated with relatively less radiation exposure than conventional radiographs such as scanograms and orthoroentgenograms. However, the CR-based teleoroentgenogram is also associated with magnification errors, and the magnitude of this error is influenced by the cassette’s distance from the X-ray source, the X-ray beam’s divergence, and the evaluated object’s length and girth. Several authors have reported a 5.0–8.8% magnification error in a CR-based teleoroentgenogram [7,13].

An AP image from a CR-based teleoroentgenogram can measure only the deviation in the coronal plane. Therefore, the analysis of rotational misalignment is performed very rarely or can only use CT images. This method delivers a high radiation dose to the patient [19,20,21]. EOS^®^ imaging is a novel low-dose biplanar digital radiographic imaging system involving highly sensitive gaseous photon detectors [22,23]. Therefore, evaluating LLD and lower limb deformities, such as rotational misalignment and fixed flexion, using the obtained double-plane 3D modeling image is possible [21]. A previous study reported that the radiation exposure of EOS^®^ image generation was 6~9 times lower than that of conventional radiographs and 4~23 times lower than that of CT [19,24]. As such, EOS^®^ imaging contributes to significant clinical and patient protection because it can simultaneously acquire and evaluate information on malformations that may occur in combination during the LLD evaluation process and reduce the risk of radiation exposure.

Escott et al. reported the magnification of each measurement method’s actual and measured lengths. A magnification error of 8.8% was found for conventional radiographs and −0.5% to −0.8% for the EOS^®^ imaging system, and they argued that the EOS^®^ was more accurate than any other measurement method [13]. Furthermore, current literature has yet to compare LLD or angles between the CR-based teleoroentgenogram and the EOS^®^ image. In this study, the magnification errors (mean ± standard deviation) when comparing both the right and left sides were 7.80 ± 1.41%, 7.3 ± 6.01%, 5.16 ± 1.25%, and 6.45 ± 0.94% for L, F, T, and F + T, respectively. The CR-based teleoroentgenogram had an average magnification of 6.8% (range, 5.2 to 7.8%), compared to the EOS^®^ imaging system in limb length. However, there was no statistical difference in the HKA angle. Although the length is magnified compared to the actual limb lengths, the HKA angle is thought to be preserved in a CR-based teleoroentgenogram. Thus, when evaluating limb length discrepancy in a CR-based teleoroentgenogram, approximately 7.8% of the magnification error should be considered. However, the magnification error is relatively negligible when evaluating angular deformity in a CR-based teleoroentgenogram. 

In a previous study comparing the relative magnification of the EOS^®^ imaging system and the CT scanogram, the EOS^®^ imaging system reported a magnification of −0.5%, and the CT scanogram reported a magnification of −1.3%. The magnification between the teleoroentgenogram and the EOS^®^ imaging system in this study was lower than 6.8%, but the EOS^®^ imaging system in the previous study was 0.8% more accurate than the CT scanogram. It can be said that the EOS^®^ imaging system is advantageous in the relative magnification accuracy, as well as the risk of radiation exposure in comparison with the CT scanogram [13].

There are some limitations to this study. First, the CR-based teleoroentgenogram and the EOS^®^ imaging measurements were not taken simultaneously. When both images were obtained, there was a possibility of changing the posture or position. Second, this study had a relatively small number of patients in subgroups. This study also focused on measuring the lengths and angles of patients, not clinical outcomes. A study with an extended replication period would make correlating the result with the clinical and functional outcome possible while providing surgeons with better suggestions in assessing limb-length discrepancies and angular deformities. 

## 5. Conclusions

The CR-based teleoroentgenogram had a magnification of about 6.8% compared to the EOS^®^ imaging system in evaluating lower extremity length. However, no difference was found in the measurement of lower limb alignment using the HKA angle. More attention to the CR-based teleoroentgenogram is needed when evaluating limb-length discrepancy than correcting angular deformities. In addition, further studies that evaluate the limb length discrepancies are necessary.

## Figures and Tables

**Table 1:** Demographic data of this study.

Variable	N (%)
Gender	
Female	56 (55.45)
Male	45 (44.55)
Age (years old)	39.39 ± 28.75: range (1–83)
Disease	
Limb length discrepancy	40 (39.60)
Angular deformity	57 (56.44)
Traumatic osteoarthritis	2 (1.98)
Baseline evaluation	2 (1.98)
Total	101 (100)

**Table 2:** Comparison of Right-Side Limb Measurements and Magnification on EOS^®^ and CR-based teleoroentgenogram.

Parameters	EOS^®^	CR Based Teleoroentgenogram	Magnification (mm)	Magnification Percentage (%)	*p* Value
Leg length [L]	669.84 ± 114.25	723.54 ± 123.52	53.7 ± 13.77	8.01 ± 1.62	<0.001 *
Femur + Tibia [F + T]	669.80 ± 116.46	715.99± 127.11	46.2 ± 28.70	6.85 ± 4.22	<0.001 *
Femur [F]	360.62 ± 62.80	391.06 ± 68.24	30.45 ± 7.36	8.44 ± 1.37	<0.001 *
Tibia [T]	309.18 ± 56.03	324.93 ± 60.40	15.75 ± 2.63	5.12 ± 0.83	<0.001 *
Hip-Knee-Ankle Angle	1.07 ± 5.10	1.40 ± 4.62			0.181

* *p* < 0.001.

**Table 3:** Comparison of Left-Side Limb Measurements and Magnification on EOS^®^ and CR- based teleoroentgenogram.

Parameters	EOS^®^	CR Based Teleoroentgenogram	Magnification (mm)	Magnification Percentage (%)	*p* Value
Leg length [L]	671.79 ± 114.19	722.92 ± 123.18	51.13 ± 11.60	7.61 ± 1.13	<0.001 *
Femur + Tibia [F + T]	667.61 ± 114.67	707.58 ± 126.68	44.34 ± 4.43	6.05 ± 5.67	<0.001 *
Femur [F]	360.21 ± 62.68	384.20 ± 76.75	23.99 ± 4.37	6.81 ± 1.14	<0.001 *
Tibia [T]	307.40 ± 52.47	323.39 ± 55.51	15.99 ± 6.30	5.20 ± 1.79	<0.001 *
Hip-Knee-Ankle Angle	1.25 ± 5.29	1.60 ± 4.96			0.66

* *p* < 0.001.

**Table 4:** Comparison of Both Right and Left-Side Limb Measurements and Magnification on EOS^®^ and CR-based teleoroentgenogram.

Parameters	EOS^®^	CR Based Teleoroentgenogram	Magnification (mm)	Magnification Percentage (%)	*p* Value
Leg length [L]	670.82 ± 113.94	723.23 ± 123.05	52.42 ± 12.76	7.80 ± 1.41	<0.001 *
Femur + Tibia [F + T]	668.7 ± 115.29	711.79 ± 126.65	43.09 ± 3.74	6.45 ± 5.01	<0.001 *
Femur [F]	360.41 ± 62.58	387.63 ± 72.52	27.21 ± 3.14	7.63 ± 1.25	<0.001 *
Tibia [T]	308.29 ± 54.15	324.16 ± 57.87	15.87 ± 1.91	5.16 ± 0.94	<0.001 *
Hip-Knee-Ankle Angle	1.16 ± 5.19	1.51 ± 4.78			0.177

* *p* < 0.001.

## Data Availability

Not applicable.
